# A COMPREHENSIVE CASE REPORT OF SEVERE PELVIC INFLAMMATORY DISEASE WITH EXTENSIVE TUBO-OVARIAN AND INTRA-ABDOMINAL ABSCESSES

**DOI:** 10.51894/001c.123085

**Published:** 2024-08-30

**Authors:** Mackenzie Miller

**Affiliations:** 1 College of Osteopathic Medicine Michigan State University

## 78

### INTRODUCTION

Pelvic inflammatory disease (PID) is an infection of the reproductive organs in women of reproductive age. PID is commonly the result of a polymicrobial infection of the vagina that through ascension affects the upper genital organs. Manifesting with symptoms ranging from lower abdominal pain and discharge to life-threatening conditions like sepsis, PID can lead to rare complications such as tubo-ovarian abscesses, Fitz-Hugh-Curtis Syndrome, and peritonitis.

### CASE DESCRIPTION

A 33-year-old female presented to the emergency department complaining of severe left lower quadrant abdominal pain, vaginal bleeding, nausea, and vomiting. The patient was seen previously and discharged after IV antibiotic treatment for similar pain and TVUS showing PID with pyosalpinx/tubo-ovarian abscess. She was unable to continue oral antibiotics and returned six days later. CT abdomen/pelvis showed 8.3 cm organized left tubo-ovarian abscess, increased in size from prior admission, 9cm right hydrosalpinx or pyosalpinx, thickened distal bowel and left colon due to peritonitis, and right quadrant loculated ascites. The abscesses increased in quantity and size throughout this hospital stay. The patient underwent IR drainage, exploratory laparotomy, and a total abdominal hysterectomy with bilateral salpingo-oophorectomy. The patient was discharged but returned with pelvic and intra-abdominal abscesses eight days later and had to be treated again with IV antibiotics.

### DISCUSSION/CONCLUSION

Pelvic inflammatory disease has a wide range of severity and complications. Due to the extensive medical and social history, discontinuous medical care, and severity of the patient’s case it resulted in one of the most severe cases of PID seen at the hospital. With the abundance of pelvic and abdominal abscesses, multiple methods of treatment were attempted including IV antibiotics, IR drainage, with the result being an exploratory laparotomy and total hysterectomy with bilateral salpingo-oophorectomy. Another factor that had to be considered through the hospital course was her age and future fertility. Along with the severity, the bacterial organisms were also unique for each admission and required her IV antibiotics to evolve throughout care. The case report shows the complexities and evolution required in a multi-admission patient with extensive pelvic inflammatory disease with tubo-ovarian and intra-abdominal abscesses.

